# Relationships between chemical structures and functions of triterpene glycosides isolated from sea cucumbers

**DOI:** 10.3389/fchem.2014.00077

**Published:** 2014-09-09

**Authors:** Joo-In Park, Hae-Rahn Bae, Chang Gun Kim, Valentin A. Stonik, Jong-Young Kwak

**Affiliations:** ^1^Department of Biochemistry, Dong-A UniversityBusan, South Korea; ^2^Department of Physiology, School of Medicine, Dong-A UniversityBusan, South Korea; ^3^Immune-Network Pioneer Research Center, Dong-A UniversityBusan, South Korea; ^4^The Laboratory of Chemistry of Marine Natural Products, G. B. Elyakov Pacific Institute of Bioorganic Chemistry, Far-Eastern Branch of the Russian Academy of ScienceVladivostok, Russia

**Keywords:** triterpene glycosides, frondoside A, cucumarioside, stichoposides, anticancer activity, membrane transporters

## Abstract

Many marine triterpene glycosides have *in vitro* and *in vivo* activities with very low toxicity, suggesting that they are suitable agents for the prevention and treatment of different diseases, particularly cancer. However, the molecular mechanisms of action of natural marine compounds in cancer, immune, and other various cells are not fully known. This review focuses on the structural characteristics of marine triterpene glycosides and how these affect their biological activities and molecular mechanisms. In particular, the membranotropic and membranolytic activities of frondoside A and cucumariosides from sea cucumbers and their ability to induce cytotoxicity and apoptosis have been discussed, with a focus on structure-activity relationships. In addition, the structural characteristics and antitumor effects of stichoposide C and stichoposide D have been reviewed along with underlying their molecular mechanisms.

## Introduction

Many marine natural products have biological activities and low toxicity suitable for administration and exhibit wide diversity in their mechanisms of action. Glycosides, substances consisting of a sugar moiety attached to a triterpene or steroid aglycone, are widely distributed in plants. Triterpene glycosides are also found in marine invertebrates and are characteristic secondary metabolites of echinoderms, octocorals, and sponges (Stonik et al., 1999; Kalinin et al., 2008; Bordbar et al., 2011).

**Figure 1:**
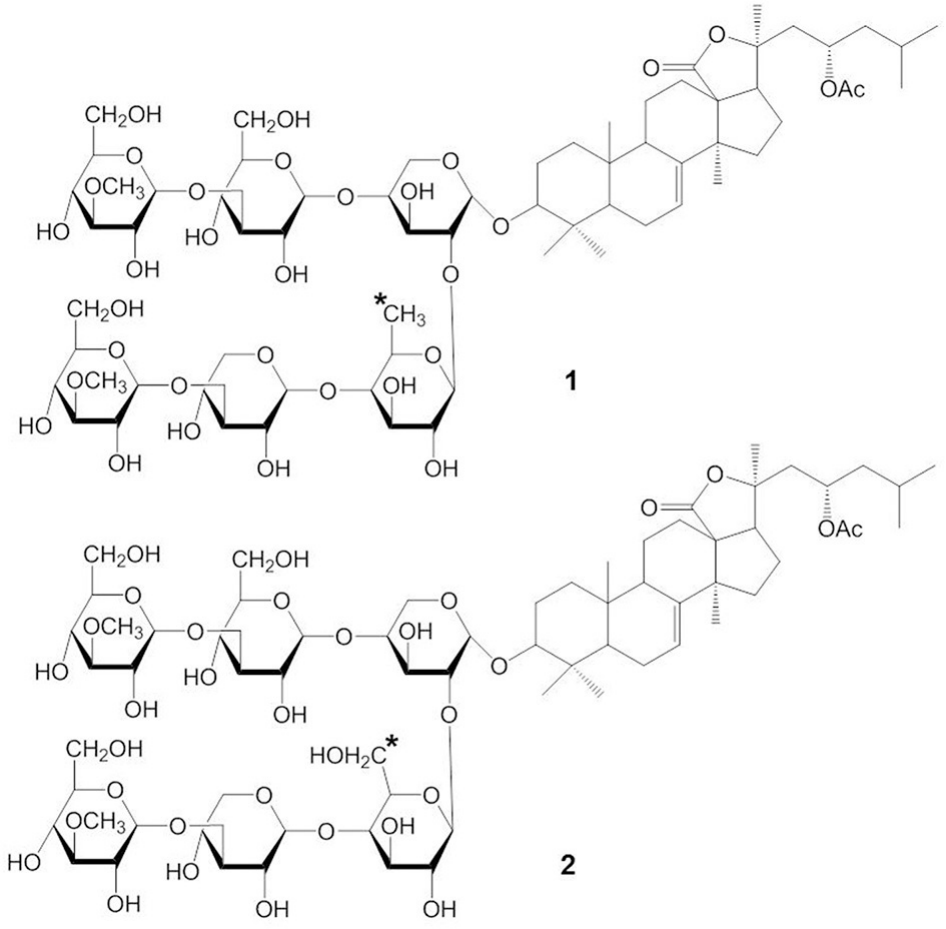
**Structures of STC (1) and STD(2)**.

**Figure 2:**
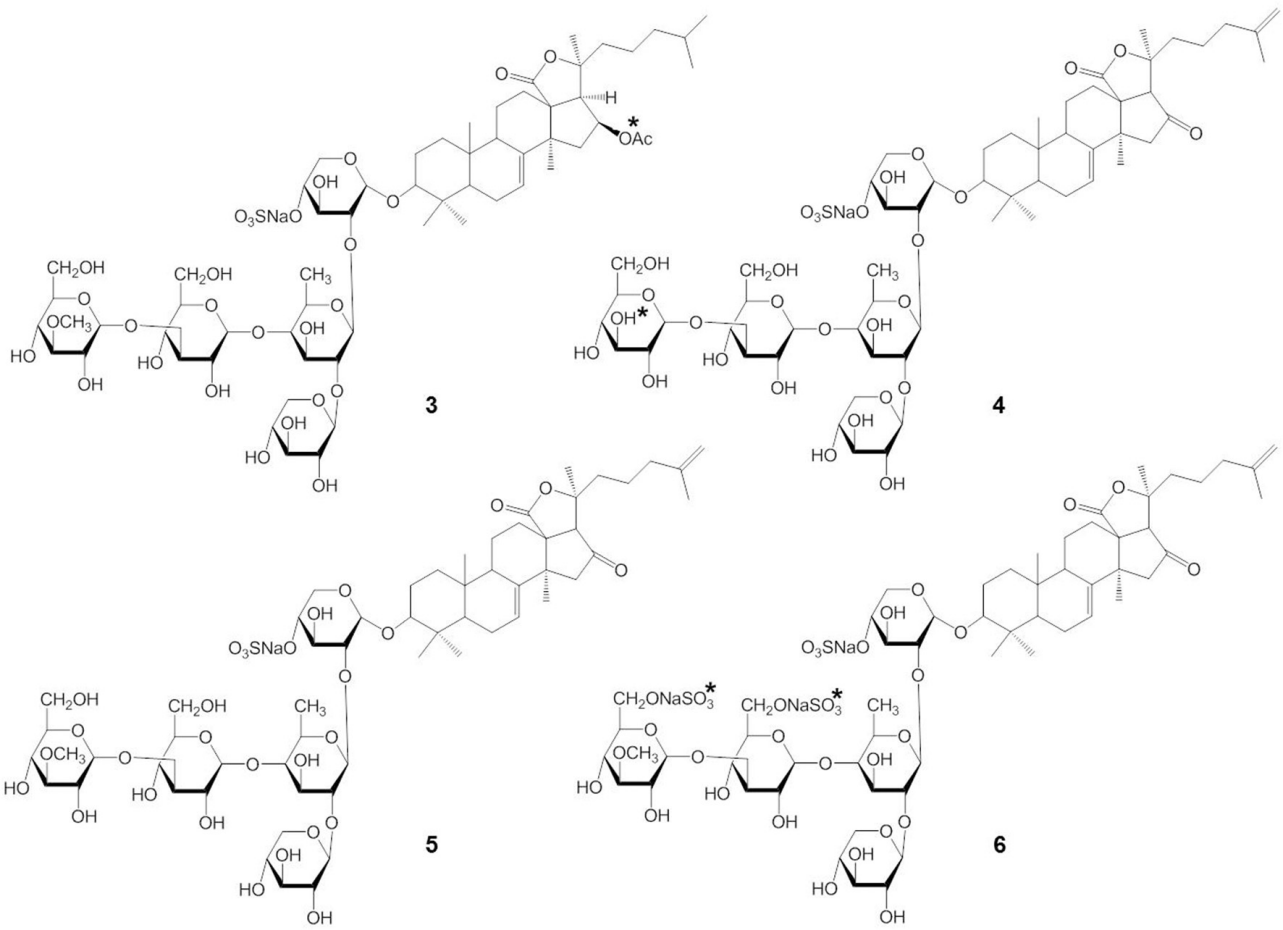
**Structures of frondoside A (3) and cucumariosides (4-6)**.

Stichoposide C (STC) (compound 1) and stichoposide D (STD) (compound 2) are hexaosides isolated from the holothurian *Stichopus chloronotus* (Figure 1) (Kitagawa et al., 1981; Stonik et al., 1982a). These compounds are also found in other representatives of the family Stichopodidae such as *Thelenota ananas* (Stonik et al., 1982b). The structural differences between STC and STD are a sugar residue; STC has quinovose, while STD has glucose as the second monosaccharide unit (indicated as the asterisk in compound 1 and 2). Frondoside A (compound 3) and cucumariosides are derived from the edible sea cucumbers *Cucumaria frondosa* and *C. japonica*, respectively (Girard et al., 1990; Stonik et al., 1999) (Figure 2). *C. japonica* is a source of several different cucumariosides such as cucumarioside A_2_-2 (compound 4), A_4_-2 (compound 5), and A_7_-1 (compound 6) (Figure 2) (Avilov et al., 1990; Drozdova et al., 1993, 1997; Stonik et al., 1999). Frondoside A and cucumariosides are pentaosides, with the main structural difference between the two compounds being the functional group at C-16 of the aglycone (acetoxy or keto group) and the third carbohydrate unit in the carbohydrate chain (indicated by an asterisk in compound 3 and 4). Interestingly, despite such similar structures, the biological activity and mechanism of frondoside A and cucumariosides appear to differ.

This review highlights the structural characteristics and mechanisms of action of marine triterpene glycosides, such as stichoposides, frondoside A, and cucumariosides. The biological activities and molecular mechanisms of several additional marine triterpene glycosides that have been studied are summarized.

## The structural characteristics of marine triterpene glycosides

**Figure 3:**
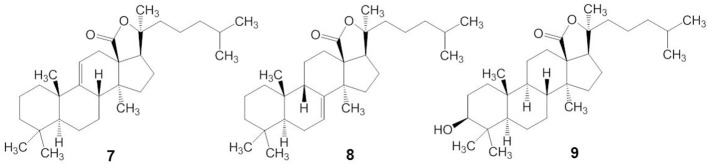
**Structures of aglycone skeleton systems with 9(11) double bond (7), 9β-H-7(8)-unsaturation (8) and 3β, 20S-Dihydroxy-5α-lanostano-18(20)-lactone (9)**.

Triterpene glycosides are the most abundant secondary metabolites in terrestrial plants and sea cucumbers. Marine triterpene glycosides are the predominant secondary metabolites of holothurians and are suggested to be responsible for their general cytotoxicity (Zhang et al., 2006a,b,c,d; Kim and Himaya, 2012; Colorado-Ríos et al., 2013). Last 20 years more than 100 new triterpene glycosides were isolated. Really, only by Russian group from Pacific Institute of Bioorganic Chemistry (PIBOC) at Vladivostok, about 30 new glycosides were isolated from *Eupentacta (Cucumaria) fraudatrix* (Silchenko et al., 2011, 2012a,b,c,d, 2013b,c), 14 new glycosides from *Cucumaria frondosa* (Girard et al., 1990; Silchenko et al., 2005a,b, 2007b), 7 from *Stuarocucumis liouvillei* (Antonov et al., 2008, 2011), 6 from *Cladolabes schmeltzi* (Silchenko et al., 2013d), 6 from *Cucumaria okhotensis* (Silchenko et al., 2007a, 2008), 5 from *Synallactes nozawai* (Silchenko et al., 2002), 5 from *Actinocucumis typica* (Silchenko et al., 2013a), 4 from *Cucumaria conicospermium* (Avilov et al., 2003), 3 from Mediterranean species (Silchenko et al., 2005c), 3 from *Pentamera calcigera* (Avilov et al., 2000a,b), 3 from *Australostichopus (Stichopus) mollis* (Moraes et al., 2005), 3 from *Achlionice violaecuspidata* (Antonov et al., 2009), 2 from *Synapta maculata* (Avilov et al., 2008), 1 from *Cucumaria koriaiensis* (Avilov et al., 1997), 1 from *Psolus eximus* (Kalinin et al., 1997), and so on. Several series of structures were also reviewed (Kalinin et al., 2012; Kim and Himaya, 2012). Reviews, completely described all the last glycosides were not yet published. Several early reviews are mentioned in our papers (Stonik et al., 1999; Kalinin et al., 2005). These are grouped into four main structural categories, based on their aglycone structures: 3β-hydroxyholost-9(11)-ene aglycone skeleton (structure 7), 9β H-3β-hydroxyholost-7-ene skeleton (structure 8), other holostane type glycosides and nonholostane glycosides (Figure 3) (Kim and Himaya, 2012).

Triterpene glycosides of holothurians typically have carbohydrates and triterpenoid moieties (Kerr and Chen, 1995; Bordbar et al., 2011). The triterpenoid moieties consist of lanostane derivatives (Zhou et al., 2005) where the majority belongs to the holostane type (Dang et al., 2007). Holostane type triterpene glycosides include a 3β, 20S-dihydroxy-5α-lanostano-18(20)-lactone structural feature (structure 9). The glycone part of natural products isolated from the sea cucumbers consists of two to six sugar units and is linked to the C-3 position of the aglycone unit (Chiludil et al., 2003; Kalinin et al., 2005). Quinovose, glucose, 3-*O*-methylglucose, xylose, and 3-*O*-methylxylose are the main sugars present in the carbohydrate moieties of these glycosides (Iñiguez-Martinez et al., 2005). In the structure of the oligosaccharide chain, the first monosaccharide unit is always a xylose, whereas 3-*O*-methylglucose or 3-*O*-methylxylose is always at the terminus. In some glycosides, sulfate groups are attached to the oligosaccharide chain. Most of these are mono-sulfated glycosides with a few occurrences of di- and tri-sulfated glycosides (Chiludil et al., 2003).

## Membranotropic and membranolytic effects of triterpene glycosides

Membranolytic effects such as increased membrane permeability, loss of barrier function, and the rupture of cell membrane are considered to be the basic mechanisms underlying a variety of biological activities exerted by triterpene glycosides from both sea cucumbers and higher plants. However, the molecular mechanisms of action of these compounds in biomembranes are not fully understood. The triterpene glycosides attach to cell membranes, interact with membrane lipids, and form glycoside-sterol complexes in biomembranes, modulating the membrane microviscosity and the activities of embedded membrane proteins (Stonik et al., 1999; Pislyagin et al., 2012). The formation of multimeric channels in sterol-containing lipid bilayers by triterpene glycosides may also be a basic mechanisms involved in increasing the permeability of membranes to ions and peptides (Li et al., 2005).

**Figure 4:**
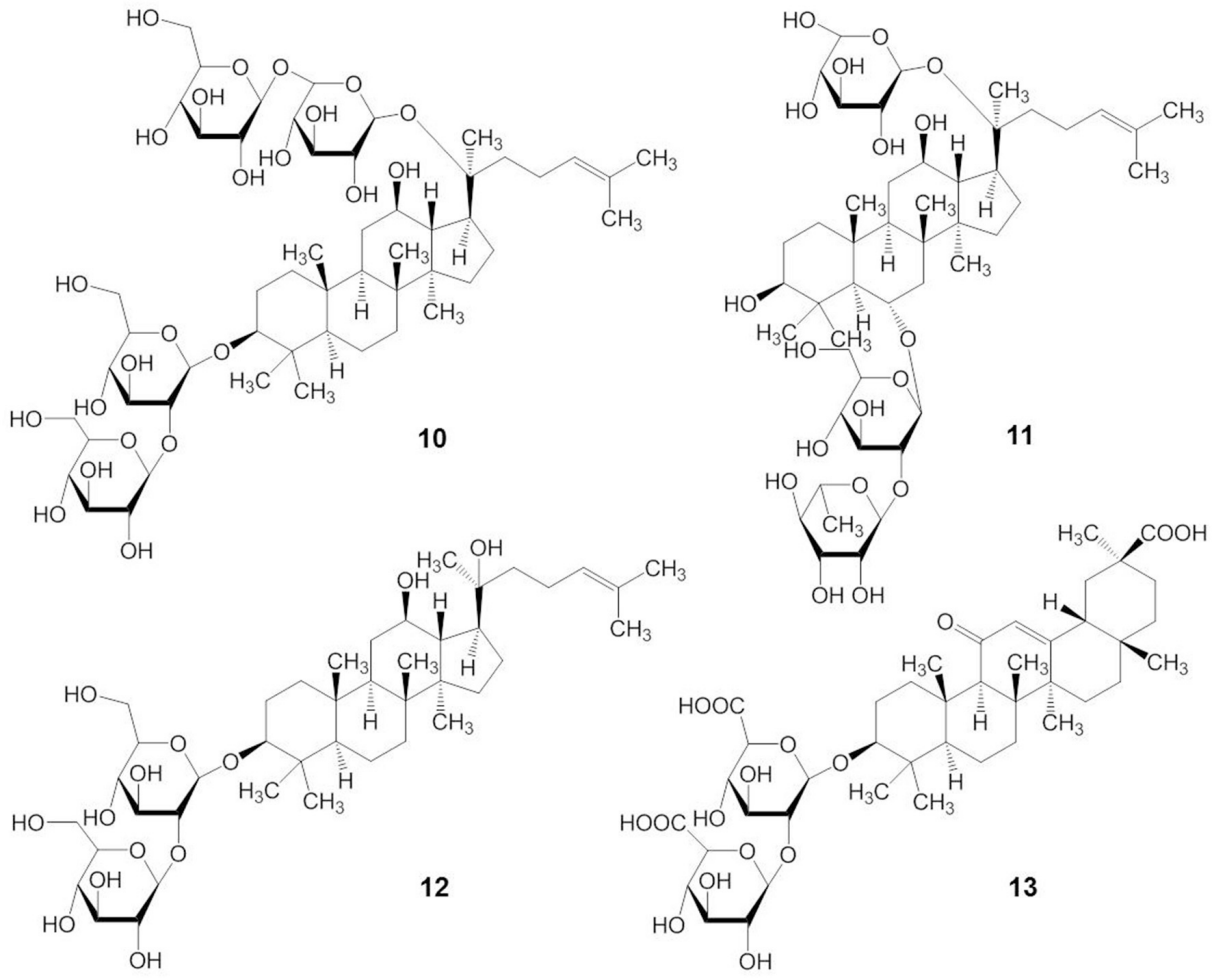
**Structures of plant triterpene glycosides**.

Although there are many subtle structural and functional differences between marine and plant triterpene glycosides, knowledge from earlier research with plant triterpene glycosides suggests that marine triterpene glycosides may have similar effects on membranes. For example, extensive studies on the membranotropic effects of plant triterpene glycosides have been performed for decades, especially with natural compounds from *Panax ginseng C.A. Meyer* (Im and Nah, 2013). The structures of some pharmacologically important plant triterpene glycosides are shown in Figure 4. Ginsenosides or ginseng saponins are major pharmacologically active ingredients of ginseng, which are composed with an aglycone of a dammarane skeleton and one or more covalently linked sugar moieties (Nah, 2014). Ginsenoside Rb_1_ has two glucopyranosyl sugar chains at C-3 and C-20 positions, respectively (compound 10) (Figure 4). Ginsenoside Re has one glucose-rhamnose disaccharide moiety at C-6 position and one glucopyranosyl moiety at C-20 position (compound 11). Ginsenoside Rg_3_ has two glucopyranosyl sugar chains only at C-3 position (compound 12). Glycyrrhizin, the main sweet-tasting constituent of licorice root, consists of a disaccharide of two glucuronic acids linked at C-3 position of the pentacyclic triterpene aglycone, glycyrrhetinic acid (compound 13). Recently, a detailed mechanism for the membrane permeabilization induced by the triterpenoid monodesmosidic saponin, α- and δ-hederin (triterpene saponins) has been proposed (Lorent et al., 2013). This mechanism includes three steps of cholesterol-independent binding to the membrane, interaction with cholesterol and asymmetric lateral distribution of saponin, and pore formation and budding of the lipid bilayer due to the increased curvature stress (Lorent et al., 2013).

More recently, at lower concentrations (in the nanomolar or low micromolar ranges) than those causing hemolytic and cytotoxic effects, the triterpene glycosides from marine sponges and sea cucumbers were found to act on specific membrane transport proteins and change their activities. For example, frondoside A and cucumarioside A_2_-2 inhibited the ATP-binding cassette (ABC) transporter, multidrug-resistance protein-1 (MDR1) (Wink et al., 2012; Menchinskaya et al., 2013). Membrane transporters which are modulated by triterpene glycosides and thus can be proposed as potential therapeutic targets are summarized in Table 1. Although the majority of researches on the membranotropic effects were done with plant triterpene glycosides, understanding the reported targets of membrane transport proteins might provide a basis for the exploration of target molecules for marine triterpene glycosides and their development as drugs.

**Table 1:** **Membrane transporters as potential targets of triterpene glycosides from sea cucumbers and plants**.

**Type**		**Membrane transporter**	**Name of compound**	**Species**	**References**
Pump		Na^+^-K^+^-ATPase	Glycyrrhizin Glycyrrhetinic acid	*Glycyrrhiza glabra*	Itoh et al., 1989
			Psolusosides A and B	*Psolus fabricii*	Gorshkova et al., 1999
		Ca^2+^-ATPase in sarcoplasmic reticulum	Cyclopiazonic acid	*Penicillium cyclopium*	Uyama et al., 1992
			Astragaloside IV	*Astragalus membranaceus*	Xu et al., 2008
		Multidrug-resistance protein-1	Saikosaponin-d	*Bupleurum falcatnum*	Wong et al., 2013
			Ginsenoside Rp_1_	*Panax ginseng*	Yun et al., 2013
			Glycyrrhizin	*Glycyrrhiza glabra*	Fu et al., 2014
			Cucumarioside A_2_-2	Cucumaria japonica^*^	Menchinskaya et al., 2013
			Frondoside A	Cucumaria okhotensis^*^	Menchinskaya et al., 2013
		Na^+^-Ca^2+^ exchange	Echinoside-A and –B	*Pearsonothuria graeffei*	Yamasaki et al., 1987
Channel	Voltage-gated	Voltage-gated Na^+^ channel	Ginsenoside Rg_3_	*Panax ginseng*	Lee et al., 2008
			Ginsenoside Rb_1_	*Panax ginseng*	Xu and Huang, 2012
			Ginsenoside Rg_3_	*Panax ginseng*	Lee et al., 2009
		Calcium-activated K^+^channel	Dehydrosoyasaponin I	*Desmodium adscendens*	McManus et al., 1993
			Ginsenoside Rg_3_	*Panax ginseng*	Choi et al., 2011a
		Human ether-a-go-go related gene K^+^ channel	Ginsenoside Rg_3_	*Panax ginseng*	Choi et al., 2011b
		L-type voltage-gated calcium channel	Ginsenoside Rb_1_	*Panax ginseng*	Lin et al., 2012
	Ligand-gated	Nicotinic acetylcholine receptor	Ginsenoside Rg_3_	*Panax ginseng*	Lee et al., 2013a
		N-methyl-D-aspartate receptor	Ginsenoside Rh_2_	*Panax ginseng*	Lee et al., 2006
			Ginsenoside Rg_3_	*Panax ginseng*	Kim et al., 2002
		GABA_A_ receptor	Ginsenoside Rg_3_	*Panax ginseng*	Lee et al., 2013b
		Ryanodine receptor	Ginsenoside Re	*Panax ginseng*	Wang et al., 2008
	Mechanosensitive	Transient receptor potential canonical	20-O-β-d-Glucopyranosyl-20(S)-protopanaxadiol	*a metabolite of ginseng saponin*	Hwang et al., 2013
	Others	Auqaporin-1	Ginsenoside Rg_3_	*Panax ginseng*	Pan et al., 2012
		Auqaporin-4	Astragaloside IV	*Astragalus membranaceus*	Li et al., 2013
Carrier		Glucose transporter (GLUT1, GLUT4)	Ginsenoside Rb_1_	*Panax ginseng*	Shang et al., 2008

* *Sea cucumbers*.

Selective inhibition of Na^+^-K^+^-ATPase and Ca^2+^-ATPase in sarcoplasmic/endoplasmic reticulum (SERCA), in combination with increased Ca^2+^ influx through L-type voltage-gated calcium channels, transient receptor potential canonical (TRPC) channels, and the ryanodine receptor led to an increase of cytosolic Ca^2+^ levels. This might explain the positive inotropic effect of triterpene glycosides (Gorshkova et al., 1999; Wang et al., 2008; Lin et al., 2012; Hwang et al., 2013; Wong et al., 2013). Furthermore, triterpene glycosides inhibited voltage-gated Na^+^ channels (Na_V_1.2 and Na_V_1.4) (Lee et al., 2008). In addition, triterpene glycosides could induce K^+^ currents through voltage-gated K^+^ channel (K_V_1.4), calcium-activated K^+^ channel (BK_Ca_), and human Ether-*à*-go-go Related Gene (hERG) K^+^ channels (Kv11.1), which might be responsible for their vasodilatory and antiarrhythmic effects (Lee et al., 2009; Choi et al., 2011a,b; Xu and Huang, 2012). The antiepileptic and neuroprotective effects of triterpene glycosides might be due to the inhibition of excitatory *N*-methyl-D-aspartate (NMDA) receptors and nicotinic acetylcholine receptors, as well as the activation of inhibitory γ-amino butyric acid (GABA) receptors (Lee et al., 2006, 2013a,b).

## Anticancer activities of marine triterpene glycosides

The antitumor action of the triterpene glycosides from sea cucumbers was discovered by Nigrelli (1952), and most of the marine triterpene glycosides that have been studied since that time are cytotoxic toward cancer cells. Nigrelli (1952) showed that injection of a “holothurin” solution inhibited the growth of Sarcoma-180 tumor cells and induced regression of the tumor. Injection of Krebs-2 ascitic tumor cells treated with holothurin into healthy mice failed to induce marked tumor growth up to 80 days (Sullivan et al., 1955). In addition, holothurin, which is a substance containing as a main constituent holothurin A, inhibited the growth of epidermal carcinoma (KB) tumor cells (Nigrelli et al., 1967).

Many triterpene glycosides from various species of sea cucumbers have diverse biological activities, including anticancer activity. For example, glycosides from 19 species of the families *Holothuriidae* and *Stichopodidae* (the glycosides in majority belong to holothurin A and B series) inhibited the growth of Sarcoma-37 cells at *in vitro* concentrations ranging from 6.2 to 100 μg/ml (Kuznetsova et al., 1982). Although the anticancer mechanisms of the triterpene glycosides have not been investigated in detail, the biologic actions, structure-activity relationships, and molecular mechanisms of stichoposide C, frondoside A, and cucumariosides have been most intensively studied (Aminin et al., 2001; Jin et al., 2009; Yun et al., 2012; Yun, 2014) and are discussed in the following sections. In addition, the potential molecular mechanisms of other triterpene glycosides have been described.

## Structure-activity relationships of marine triterpene glycosides

**Figure 5:**
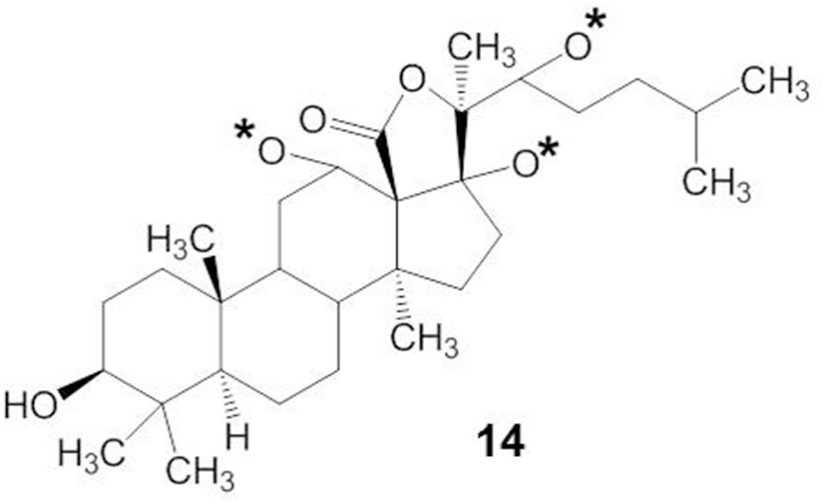
**Structures of 18(20)-lactone in the aglycone with oxygen group**.

The molecular mechanisms of action of marine triterpene glycosides can be understood by uncovering the relationships between their structure and activities. However, the structure-activity relationships of marine triterpene glycosides have not been intensively studied. As shown in structure 14, the presence of an 18(20)-lactone in the aglycone, with at least one oxygen group nearby (indicated by an asterisk in structure 14), is significant for the biological activity of triterpene glycosides bearing a 9(11)-double bond (Kitagawa, 1988) (Figure 5). Glycosides that have a 7(8)-double bond in their aglycone without a 16-keto group are more active in hemolytic test than those with a 16-keto group (Kalinin et al., 1996). In general, the characteristics of the attached glycone structure may be related to the biological activities of the marine triterpene glycosides.

**Figure 6:**
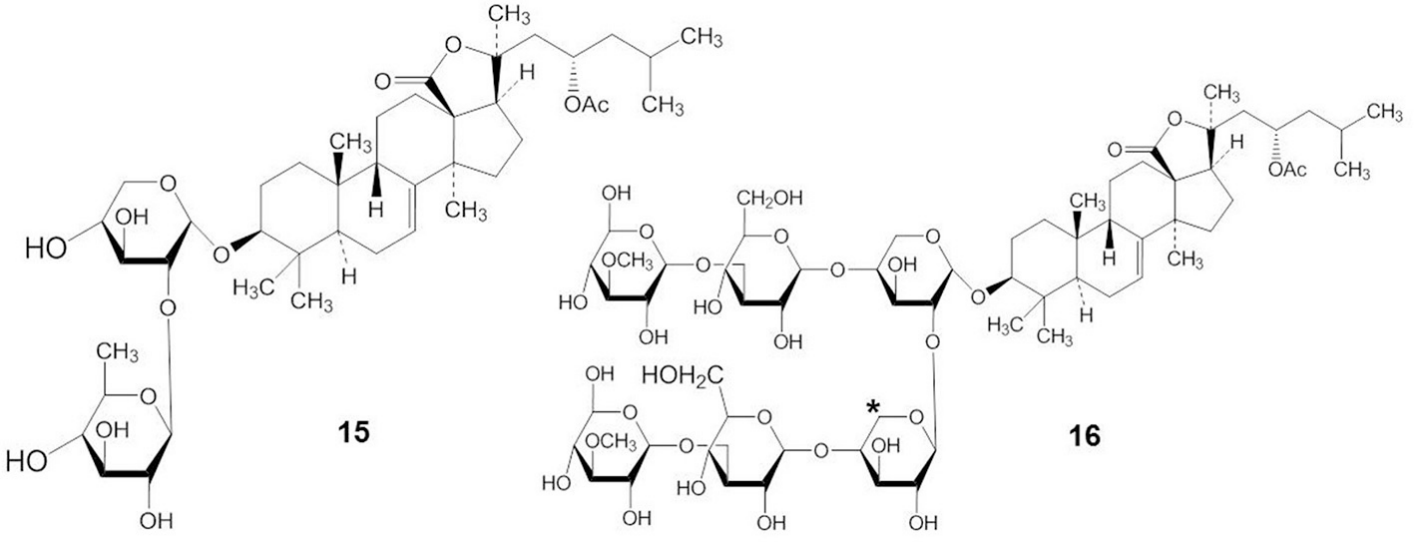
**Structures of STA (15) and STE (16)**.

Many investigators have suggested that the bioactivity of the triterpene glycosides results from their strong membranolytic activity. It was reported that the membranolytic activity of triterpene glycosides was due to the formation of complexes between the glycosides and the 5(6)-unsaturated sterols within target cellular membranes (Kalinin, 2000). A linear tetrasaccharide chain of triterpene glycosides is necessary for the effects leading to modification of the cellular membrane (Kitagawa, 1988; Kalinin et al., 1992). Stichoposide A (STA) (compound 15), which had two monosaccharide units, and stichoposide E (STE) (compound 16), which has a xylose residue as the second monosaccharide unit (indicated by an asterisk in compound 16), had lesser membranotropic activity than other stichoposides (Kalinin et al., 2008) (Figure 6). Maltsev et al. (1985) reported that glycosides with quinovose as the second monosaccharide unit were more active hemolytics than other triterpene glycosides.

**Figure 7:**
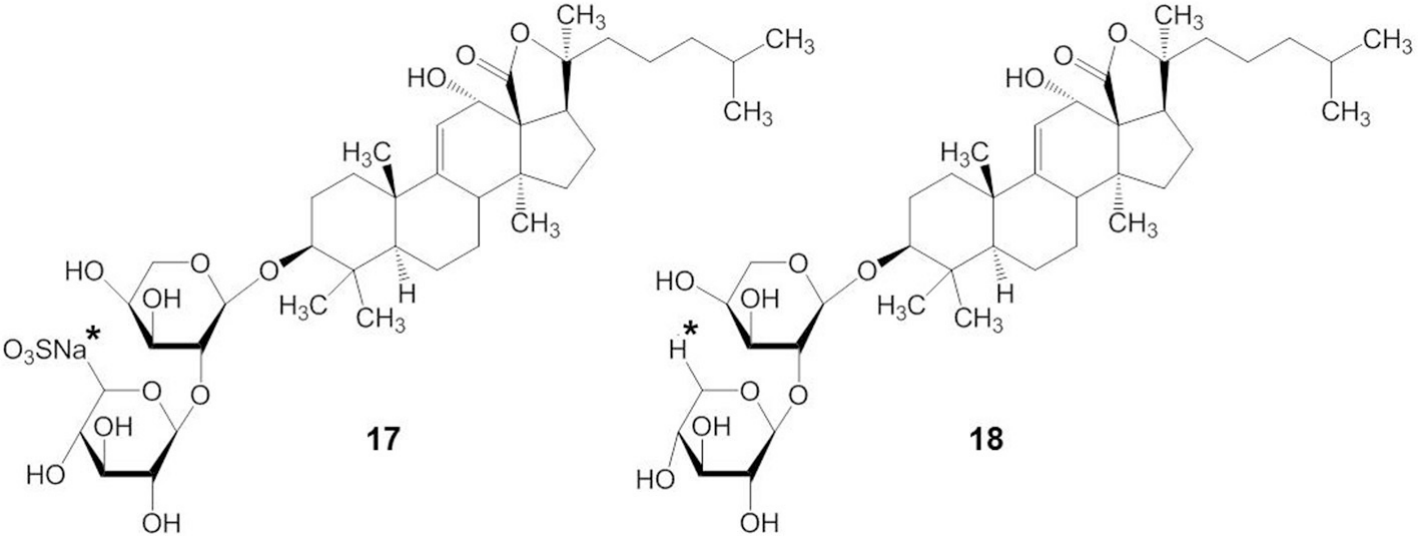
**Structure of compound with (17) or without (18) a sulfate group at C-4 of the xylose residue**.

The presence or absence of a sulfate group in the sugar chain of triterpene glycosides influences their bioactivity (Kalinin, 2000; Kim and Himaya, 2012). A sulfate group at C-4 of the first xylose of non-branched glycosides with a linear tetrasaccharide unit (compound 17) does not significantly affect the activity of triterpene glycosides, but the absence of a sulfate group at C-4 of the xylose residue (compound 18) decreases their activity (indicated by an asterisk in compound 17 and 18) (Figure 7) (Kitagawa, 1988; Kalinin et al., 1992). In contrast, the presence of a sulfate at C-4 of the first xylose in branched pentaosides with 3-*O*-methyl group on the terminal monosaccharide increases their activities, while the same sulfate decreases the activity of branched pentaosides that have glucose as the terminal residue. Sulfate groups attached to the C-6 position of terminal glucose or 3-*O*-methylglucose residues in triterpene glycosides greatly reduce their activity (Kalinin, 2000; Kim and Himaya, 2012).

## Stichoposides

### STC

STC (also called stichloroside C1) is a quinovose-containing hexaoside, originally isolated from the holothurian *S. chloronotus* (Kitagawa et al., 1981; Stonik et al., 1982a) but is also found in other representatives of the family *Stichopodidae* such as *T. ananas* (Stonik et al., 1982b). STC has quinovose as the second monosaccharide unit. The antitumor activity of STC appears to be related to its membranotropic effects (Kalinin et al., 2008). We previously reported that STC induced apoptosis of human leukemia and colorectal cancer cells through the activation of both intrinsic and extrinsic pathways (Yun et al., 2012). Anticancer agents increase ceramide levels, to variable extents, in all types of cancer cells (Taha et al., 2006). Ceramide is generated either by *de novo* synthesis or by sphingomyelin hydrolysis through the action of several types of sphingomyelinase (SMase) such as acid, neutral, or alkaline SMase (Strum et al., 1997; Brown and London, 1998; Kolesnick et al., 2000; Hannun and Obeid, 2008). Both acid and neutral SMase are involved in ceramide generation in response to apoptotic stimuli (Levade and Jaffrezou, 1999; Goni and Alonso, 2002; Gulbins and Kolesnick, 2002). Moreover, under conditions where the classical apoptotic pathway fails, intracellular generation of ceramide may function as part of a backup system that enables caspase-independent programmed cell death (Taha et al., 2006). We demonstrated that STC induced apoptosis through the generation of ceramide by the activation of acid and neutral SMases (Yun et al., 2012). Therefore, the target of STC seems to be SMase leading to increases in ceramide and apoptosis.

### STD

STD is a hexaoside containing glucose at the second monosaccharide unit. We have shown that STD can induce apoptosis of human leukemia cells via the extrinsic and intrinsic pathways (Park et al., 2012a). We previously compared the potency of STC and STD in the induction of apoptosis using human leukemia K562 and HL-60 cells. STC was two to five times more potent than STD in inducing cell death [IC_50_ = 0.5 (K562 cells) and 0.3 (HL-60 cells) μM for STC; 1.0 (K562 cells), and 1.5 (HL-60 cells) μM for STD] (Park et al., 2012a). These results are consistent with the relative membranotropic activities of STC and STD, suggesting that their anticancer activities may be related to their membranotropic activities. More importantly, STC and STD did not have any toxicity in normal hematopoietic progenitor cells or in a mouse tumor model (Yun et al., 2012; Yun, 2014).

It was shown that STD induced apoptosis by activating ceramide synthase 6 (CerS6) and increasing cellular levels of ceramide (Yun, 2014). The activation of CerS6 appears to be subsequent to the activation of the death receptor Fas (CD95) by STD (Yun, 2014). These results suggest that the difference in only one sugar between STC and STD may influence both the potency and the molecular mechanisms for their activities. However, further studies on the relationship between the structure and the activity of these molecules are needed to improve the efficacy and safety of these compounds in treating cancer patients.

## Frondoside A and cucumariosides

### Biological actions of frondoside A and the cucumariosides

Frondoside A has a sulfate, an acetoxy group at C-16 of the aglycone, penta-saccharide chain with xylose as the third monosaccharide residue, and 3-*O*-methylglucose as the terminal monosaccharide residue (Girard et al., 1990). Cucumarioside A_4_-2 has a 16-keto group in the aglycone and a glucose residue as the third monosaccharide unit in the carbohydrate chain (Kalinin et al., 1992, 1996). Cucumarioside A_2_-2 has 3-*O*-methylglucose instead of glucose as the terminal monosaccharide unit. Cucumarioside A_2_-2 is probably biogenetically connected with A_4_-2 (Kalinin et al., 1992). Therefore, the main structural differences between frondoside A and the cucumariosides, as shown in Figure 2, are in a functional group at C-16 of the aglycone and the third carbohydrate unit in the carbohydrate chain.

Frondoside A and cucumariosides show anticancer activities *in vitro* and suppress tumor growth *in vivo* (Tian et al., 2005; Tong et al., 2005; Li et al., 2008). The antitumor activity of frondoside A and cucumariosides is a result of their activity to induce apoptosis of cancer cells (Li et al., 2008; Jin et al., 2009; Roginsky et al., 2010), including HL-60, NB-4, and THP-1 leukemic cells (Jin et al., 2009).

The cancer inhibitory effect of frondoside A in tumor-bearing mice might partly result from other biological activities, including its antiangiogenic and antimetastatic effects (Li et al., 2008; Al Marzouqi et al., 2011; Ma et al., 2012; Attoub et al., 2013). In addition, frondoside A inhibited the invasion of breast cancer cells via its ability to decrease matrix metalloproteinase (MMP)-9 expression through inhibition of nuclear translocation and transactivation of NF-κB and AP-1 (Park et al., 2012b). Park et al. (2012b) also showed that frondoside A significantly inhibited PI3K/Akt, ERK-1/2, and p38 MAPK activation in 12-*O*-tetradecanoyl-phorbol-13-acetate (TPA)-stimulated breast cancer cells, indicating that frondoside A inhibited TPA-induced NF-κB and AP-1 activation via inactivation of the PI3K/Akt, ERK1/2 and p38 MAPK pathways. Frondoside A also decreased AP-1-dependent transcriptional activities in JB6-LucAP-1 cells (Silchenko et al., 2008).

It is well established that prostaglandin E receptor, EP_4_ that is expressed in a number of different malignancies, can promote the migration of tumor cells *in vitro* (Timoshenko et al., 2003; Wang and Dubois, 2010). EP_4_ also promotes the invasive behavior of inflammatory breast cancers, one of the more aggressive forms of breast cancers (Robertson et al., 2010). Frondoside A inhibited metastasis of breast cancer cells by antagonizing EP_4_ and EP_2_ (Ma et al., 2012).

Cucumariosides increased the lysosomal activity and intracellular Ca^++^ concentrations of macrophages. These effects are related to the chemical structures of the molecules. For example, although there was no direct correlation, Silchenko et al. (2013c) suggested that the lysosomal activity and cytotoxicity of cucumarioside depended on features of both the aglycone and the carbohydrate chain. Holt et al. (2012) have investigated the effect of frondoside A on NK cells and demonstrated that prostaglandin E_2_ (PGE_2_) significantly suppressed the secretion of interferon-γ (IFNγ) from NK cells while frondoside A restored the capacity of NK cells to secrete IFNγ in the presence of PGE_2_. Other studies reported that *in vitro* treatment of peritoneal macrophages with cucumarioside A_2_-2 stimulated cell adhesion as well as their spreading reaction and motility (Aminin et al., 2011), whereas frondoside A suppressed MMP-9 enzymatic activity, secretion, and expression in MBA-MB-231 human breast cancer cells, leading to inhibition of invasion and migration of these cells (Park et al., 2012b). Therefore, it is important to compare the effects of frondoside A and cucumariosides on the migration and spreading of various kinds of cells, including cancer and immune cells.

### Effects of sulfate groups on the hemolytic activity of cucumariosides

The structures of the aglycone and carbohydrates in cucumariosides may confer membranolytic activity (Stonik et al., 1999). Kalinin et al. (1996) demonstrated that the membranolytic properties of cucumariosides correlated with their cytotoxicity to tumor cells. Cucumarioside A_2_-2 had *in vitro* cytotoxic and hemolytic effects on sea urchin embryos with EC_50s_ of 0.45 and 5 μg/mL, respectively (Aminin et al., 2006, 2010). The LD_50_ of cucumarioside A_2_-2 for mice was 10 mg/kg after intraperitoneal injection (Polikarpova et al., 1990). The membranolytic action of cucumariosides may be mediated through formation of molecular complexes with sterols in membranes and subsequent generation of solitary ion channels and large aqueous pores (Anisimov, 1987; Verbist, 1993; Kalinin et al., 2008). In addition, the glycosides effectively increased the microviscosity of the lipid bilayer of cell membranes (Pislyagin et al., 2012).

Marine triterpene glycosides contain different numbers of sulfate groups bound with sugars. Cucumarioside A_2_-2 has a sulfate group at C-4 of the first xylose residue and cucumarioside A_6_-2 has an additional sulfate group at C-6 of the terminal 3-*O*-methylglucose residue. The hemolytic activity of cucumarioside A_2_-2 was significantly greater than its desulfated derivative and was higher than that of cucumarioside A_6_-2 (Kalinin et al., 1996). Moreover, cucumarioside A_2_-2 had more active hemolytic activity than cucumarioside A_3_, which has an additional sulfate group at C-6 of the third monosaccharide unit (Kalinin et al., 1996). The increase in intracellular Ca^2+^ concentrations was also influenced by the number and positions of sulfate groups in the carbohydrate moiety of the molecules. Cucumarioside A_2_-2 was more active in inducing a rapid increase in cytosolic Ca^2+^ content, when compared to the poly-sulfated derivative of A_2_-2, cucumarioside A_7_-1 (indicated by an asterisk in compound 5) (Agafonova et al., 2003). In addition, the mono-sulfated cucumariosides A_2_-2 and A_4_-2 stimulated peritoneal macrophage lysosomal activity, while desulfation of their carbohydrate moiety completely abolished this activity (Aminin et al., 2001). Therefore, the hemolytic and cytotoxic activities of triterpene glycosides may be dependent on the positions of sulfate groups attached to the carbohydrates.

### Cytotoxic effects of frondoside A and cucumariosides on cancer cells

Frondoside A showed potent cytotoxicity against various cancer cells, including HeLa, HL-60, and lung cancer cells such as LNM35, A549, and NCI-H460-Luc2 (Silchenko et al., 2008; Jin et al., 2009; Attoub et al., 2013). Moreover, frondoside A enhanced the inhibition of lung tumor growth induced by the anticancer agent, cisplatin (Attoub et al., 2013). The IC_50_ of frondoside A in HL-60 cells was approximately 5- to 10- fold lower than that of cucumarioside A_2_-2 (Jin et al., 2009), although the *in vivo* toxicity of these two compounds for mice was similar (Polikarpova et al., 1990; Aminin et al., 2001). Overall, the structures of both the aglycone and the carbohydrate chain seem to be very important for the cytotoxic activity of frondoside A and the cucumariosides against cancer cells. However, some changes in the carbohydrate residues may not play a significant role in the cytotoxicity of triterpene glycosides because the cucumarioside A_2_-2 and cucumarioside A_4_-2 differ only in the structure of their terminal monosaccharide residue having glucose and methylglucose, respectively.

**Figure 8:**
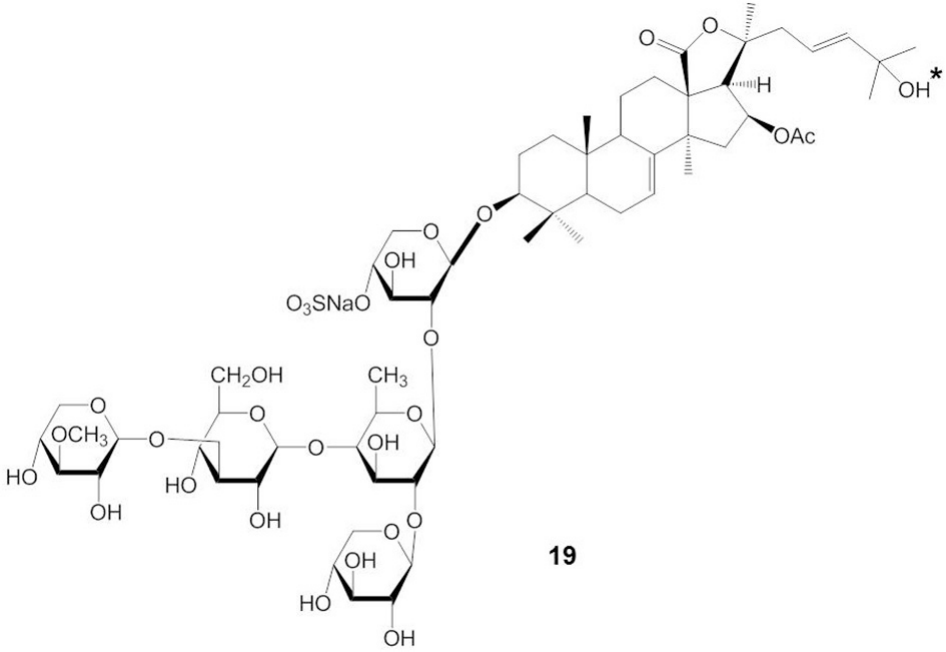
**Structure of cucumarioside H_2_ having 25-OH group**.

Silchenko et al. (2012d) suggested that amphiphilicity might affect the cytotoxic potency of cucumarioside by demonstrating that the presence of a 25-OH group in the aglycone moiety of triterpene glycosides (cucumarioside H_2_) (indicated by an asterisk in compound 19) significantly decreased their cytotoxicity, but the cucumarioside having 25-ethoxy group (cucumarioside H_4_) had potent cytotoxic activity against lymphocytes and very high hemolytic activity (Figure 8). Our study suggested that the acetyl group at C-16 of the aglycone moiety might play a significant role in the cytotoxicity of triterpene glycosides because frondoside A had more potent effects than cucumarioside A_2_-2 (Jin et al., 2009). In contrast, the presence of acetyl groups in steroids increased their cytotoxic potency (Mimaki et al., 2001).

### Apoptotic effects of frondoside A and cucumariosides on cancer cells

Frondoside A caused a concentration-dependent reduction in the viability of lung cancer cells (LNM35, A549, and NCI-H460-Luc2), melanoma cells (MDA-MB-435), breast cancer cells (MCF-7), and hepatoma cells (HepG2) over 24 h, and increased the activities of caspases-3 and -7 in LNM35 lung cancer cells (Attoub et al., 2013). It was also shown that treatment of human pancreatic cancer cells with a low concentration of frondoside A induced apoptosis through increased activities of caspases-9, -3, and -7, increased bax, and decreased bcl-2 and mcl-1 (Li et al., 2008). Our results demonstrated that mitochondrial membrane permeability was not changed, and the accumulation of cytochrome c in the cytosolic fraction was not observed in HL-60 cells treated with frondoside A (Jin et al., 2009). Similarly, frondoside A had more potent effects than cucumarioside A_4_-2 on apoptosis in leukemic cells but did not induce caspase activation before early apoptosis, whereas cucumariosides A_2_-2 and A_4_-2 showed similar effects on pro-caspase cleavage and mitochondrial permeability (Jin et al., 2009). Moreover, the annexin-V positivity induced by frondoside A was not inhibited by the pancaspase inhibitor, zVAD-fmk, whereas both the annexin-V positivity and cleavage of caspases induced by cucumarioside A_2_-2 were efficiently blocked by zVAD-fmk. These results suggest that frondoside A initiates apoptosis in a caspase-independent manner in some cancer cells. Determination of the structural characteristics responsible for the differential effects of frondoside A and cucumariosides on inducing apoptosis in cancer cells will be essential to reveal their mechanisms of action.

## Other marine triterpene glycosides

Previous studies demonstrated that marine triterpene glycosides from sea cucumbers had anticancer activities (Stonik, 1986; Stonik et al., 1999). However, the molecular mechanisms for their anticancer activities were only partly defined. Here, we briefly review potential molecular mechanisms for the anticancer activity of several marine triterpene glycosides. A summary of this information is shown in Table 2.

**Table 2:** **Potential molecular mechanisms for anticancer activity of marine triterpene glycosides**.

**Name**	**Species**	**Actions**	**Molecular mechanisms**	**IC_50_**	**References**
Frondoside A	*Cucumaria frondosa*	Inhibition of proliferation	Increased expression of p21	4 μg/mL (AsPC-1 cells)	Li et al., 2008
		Induction of apoptosis	Caspase-independent pathway, mitochondrial pathway, increased expression of p53	1 μM (HL-60 cells)	Jin et al., 2009
				2. 5 μM (MDA-MB 231 cells)	
			Decreased expression of Bcl-1 and Mcl-1, increased expression of Bax	4 μg/mL (AsPC-1 cells)	Al Marzouqui et al., 2011
		Antimetastatic activity	Inhibition of MMP-9 activation	1 μM (MDA-MB-231 cells)	Li et al., 2008
			Inhibition of prostaglandin receptors EP_4_ and EP_2_	0.5 μM (Line 66.1 cells)	Park et al., 2012b
					Ma et al., 2012
Stichoposide C	*Thelenota anax*	Induction of apoptosis	Extrinsic and intrinsic pathway, activation of acid SMase and neutral SMase, ceramide generation	0.3 μM (HL-60 cells)	Yun et al., 2012
				0.5 μM (K562 cells)
Stichoposide D	*Thelenota anax*	Induction of apoptosis	Extrinsic and intrinsic pathway, activation of ceramide synthase 6, ceramide generation	1.5 μM (HL-60 cells)	Park et al., 2012a
				1.0 μM (K562 cells)	Yun, 2014
Cucumaioside A_2_-2, A_4_-2	*Cucumaria japonica*	Induction of apoptosis	Caspase-dependent pathway	3 μM (HL-60 cells)	Jin et al., 2009
Echinoside A	*Holothuria nobilis*	Induction of apoptosis	Inhibition of the noncovalent binding of topoisomerase 2α to DNA	2.4 μM (human cancer cell lines)	Li et al., 2010
	*Peasonothuria graeffei*	Cell cycle arrest	Increased expression of *p16, p21, and c-myc*, decreased expression of cyclin D1	2.7 μM (HepG2 cells)	Zhao et al., 2012
Ds-echinoside A	*Peasonothuria graeffei*	Antimetastatic activity	Inhibition of NF-κB dependent MMP-9 and VEGF expression	2.7 μM (HepG2 cells)	Zhao et al., 2011
Philinopside A	*Pentacta quadrangularis*	Induction of apoptosis	Inhibition of receptor tyrosine kinase autophosphorylation	1.5−2.4 μM (Sarcoma 180, BEL-7402, MCF-7 cells)	Tong et al., 2005
Philinopside E	*Pentacta quadrangularis*	Antimetastatic activity	Inhibition of VEGFR2 signaling	~4 μM	Tian et al., 2005
			Inhibition of interaction between KDR and α_v_β_3_ integrin	2.5 μM	Tian et al., 2007

### Echinoside A and DS-echinoside A

**Figure 9:**
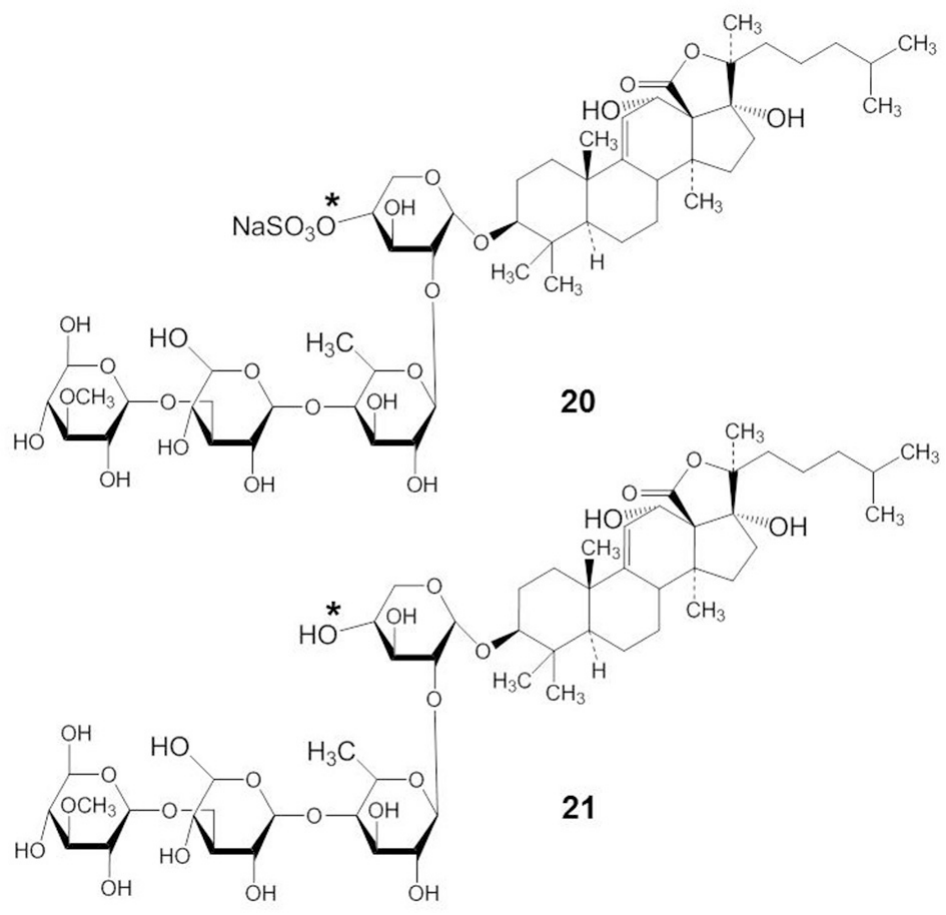
**Structures of EA (20) and DSEA (21)**.

Echinoside A (EA) and DS-echinoside A (DSEA) belong to the holostane glycoside type with an 18(20)-lactone. Both have identical aglycone structures and there are only small structural differences in their carbohydrate chains. EA has a sulfate group at C-4 of the first xylose residue (compound 20) but DSEA is a non-sulfated triterpene glycoside (compound 21) (indicated by an asterisk in compound 20 and 21) (Figure 9). Li et al. (2010) have shown that EA, which was isolated from the sea cucumber *Holothuria nobilis*, displayed potent anticancer activities through inhibition of the noncovalent binding of topoisomerase 2α to DNA, resulting in double strand breaks and subsequent cell apoptosis. EA is the first marine-derived topoisomerase inhibitor identified with a saponin skeleton. Zhao et al. (2012) demonstrated that EA, isolated from *Pearsonothuria graeffei*, inhibited cell proliferation by arresting the cell cycle in the G_0_/G_1_ phase and inducing apoptosis, with DSEA exerting the strongest effect. DSEA exhibited more potent anticancer activity than EA. This suggests that the sulfate group at C-4 of the first xylose residue may reduce the anticancer activity of EA. Zhao et al. (2011) demonstrated that DSEA, isolated from the sea cucumber *Pearsonothuria graeffei*, inhibited the main steps involved in metastasis of HepG2 cells, including suppression of cell migration, adhesion, and invasion. DSEA suppressed MMP-9 and VEGF expression and enhanced the expression of TIMP by blocking the NF-κB signaling pathway in a dose-dependent manner. This indicated that a desulfation reaction at xylose C-2 might be related to NF-κB targeting in tumor metastasis.

### Philinopside A and E

**Figure 10:**
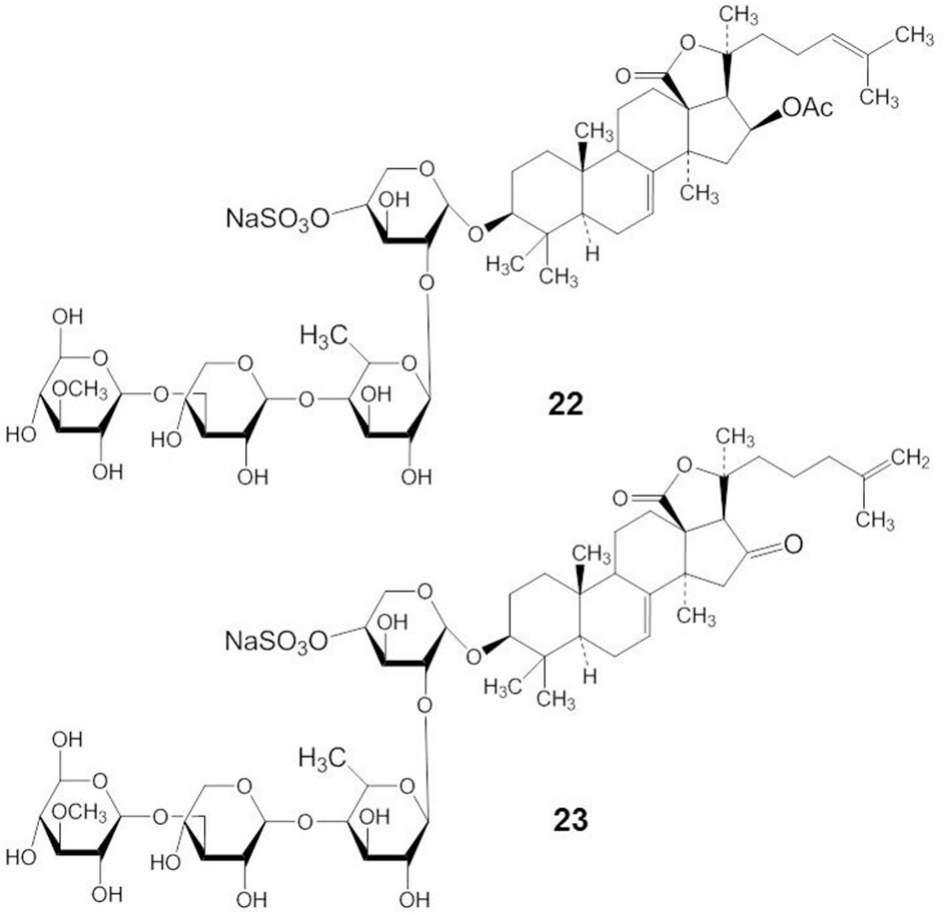
**Structures of PA (22) and PE (23)**.

Philinopside A (PA) (compound 22) is a compound isolated from the sea cucumber *Pentacta quadrangularis* (Figure 10). PA exhibited antitumor effects both *in vitro* and *in vivo* through the inhibition of autophosphorylation of receptor tyrosine kinases, including growth factor receptor, platelet derived growth factor receptor-β, and fibroblast growth factor receptor (Tong et al., 2005).

Philinopside E (PE) (compound 23) is a new sulfated saponin from sea cucumbers. PE inhibits cell adhesion, migration, and invasion through the inhibition of vascular endothelial growth factor receptor 2 (VEGFR2) signaling leading to the suppression of Akt, ERK, focal adhesion kinase, and paxillin (Tian et al., 2005). In addition, Tian et al. (2007) have shown that PE specifically interacted with the extracellular domain of the kinase insert domain-containing receptor (KDR) to block its interaction with VEGF and inhibited downstream signaling. More specifically, PE markedly suppressed α_v_β _3_ integrin-driven downstream signaling as a result of disturbing the physical interaction between KDR and α_v_β_3_ integrin in human microvascular endothelial cells, followed by disruption of the actin cytoskeleton organization and decreased cell adhesion to vitronectin (Tian et al., 2007).

## Conclusions

Sea cucumbers contain physiologically active triterpene glycosides. Biological effects, including anticancer activities of several marine triterpene glycosides are observed *in vitro* and *in vivo*. Research regarding the mechanisms of action of marine triterpene glycosides on membrane transporters is very limited despite extensive studies on similar compounds in plants. Taking into account the structural and functional differences between marine and plant triterpene glycosides, more intensive studies are required with natural marine triterpene glycosides to assess their potential as novel drugs for the treatment of diseases, including cancer. The STC has anticancer activity through the generation of ceramide by a different mechanism from STD because of the sugar moiety. The anticancer effects of frondoside A and cucumariosides might be through inhibition of tumorigenesis and metastasis and modulation of antitumor immune responses. However, both frondoside A and cucumariosides also possess membranolytic, cytotoxic, and apoptotic properties with different potencies and mechanisms. Structural differences between frondoside A and cucumarioside seem to be responsible for their different biological activities. Thus, identification of the structural characteristics controlling the biological activities of marine triterpene glycosides is essential for developing marine drugs.

### Conflict of interest statement

The authors declare that the research was conducted in the absence of any commercial or financial relationships that could be construed as a potential conflict of interest.
