# Corrigendum: Relationships between chemical structures and functions of triterpene glycosides isolated from sea cucumbers

**DOI:** 10.3389/fchem.2014.00103

**Published:** 2014-11-13

**Authors:** Jong-Young Kwak

**Affiliations:** Department of Biochemistry, School of Medicine, Dong-A UniversityBusan, South Korea

**Keywords:** anticancer activity, cucumarioside, frondoside A, membrane transporters, stichoposides, triterpene glycosides

The authors corrected structures of compounds in Figures 2, 4, 6, and 9.

**Figure 2:**
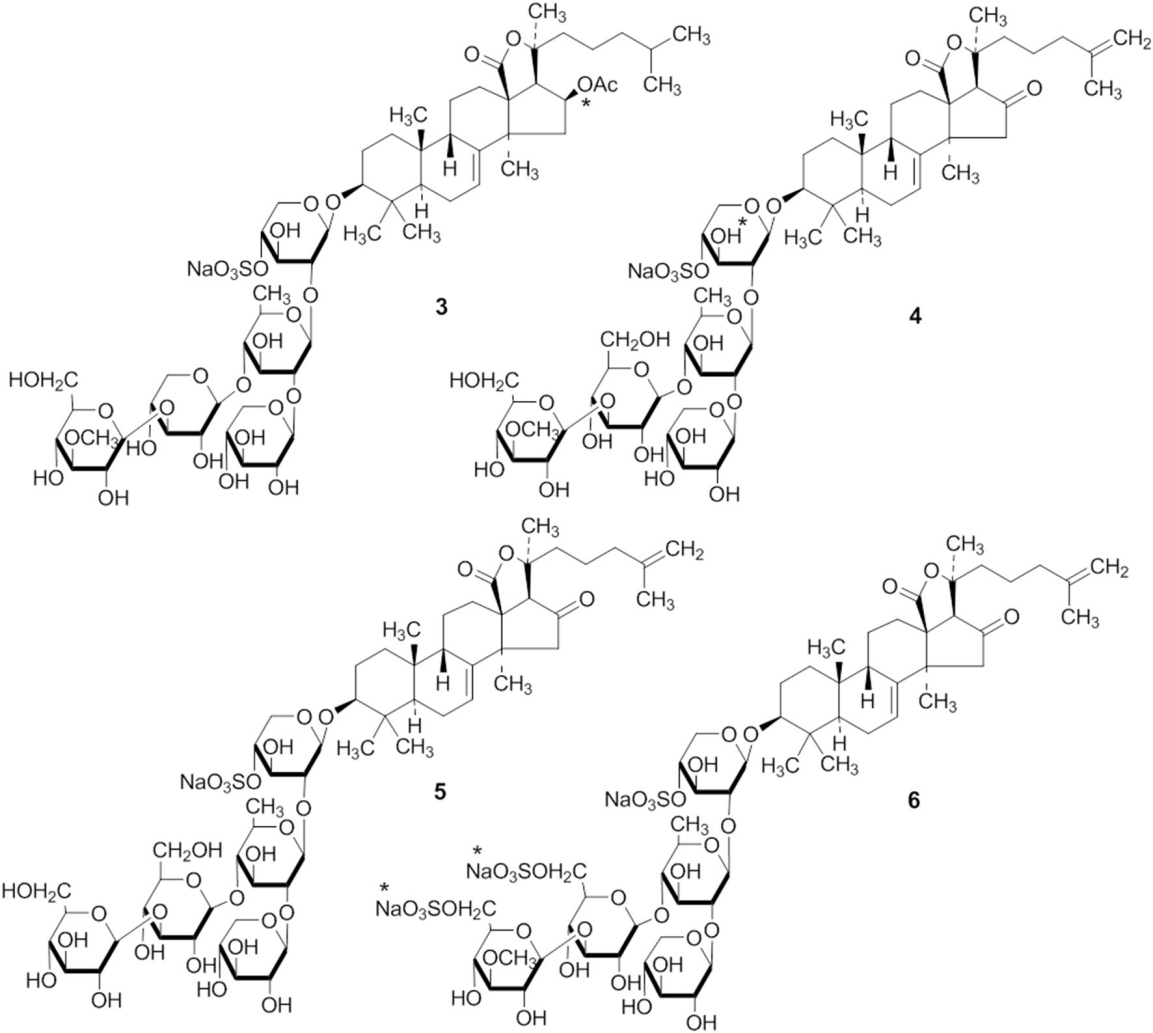
**The structures of 3, 4, 5 and 6 are corrected**.

**Figure 4:**
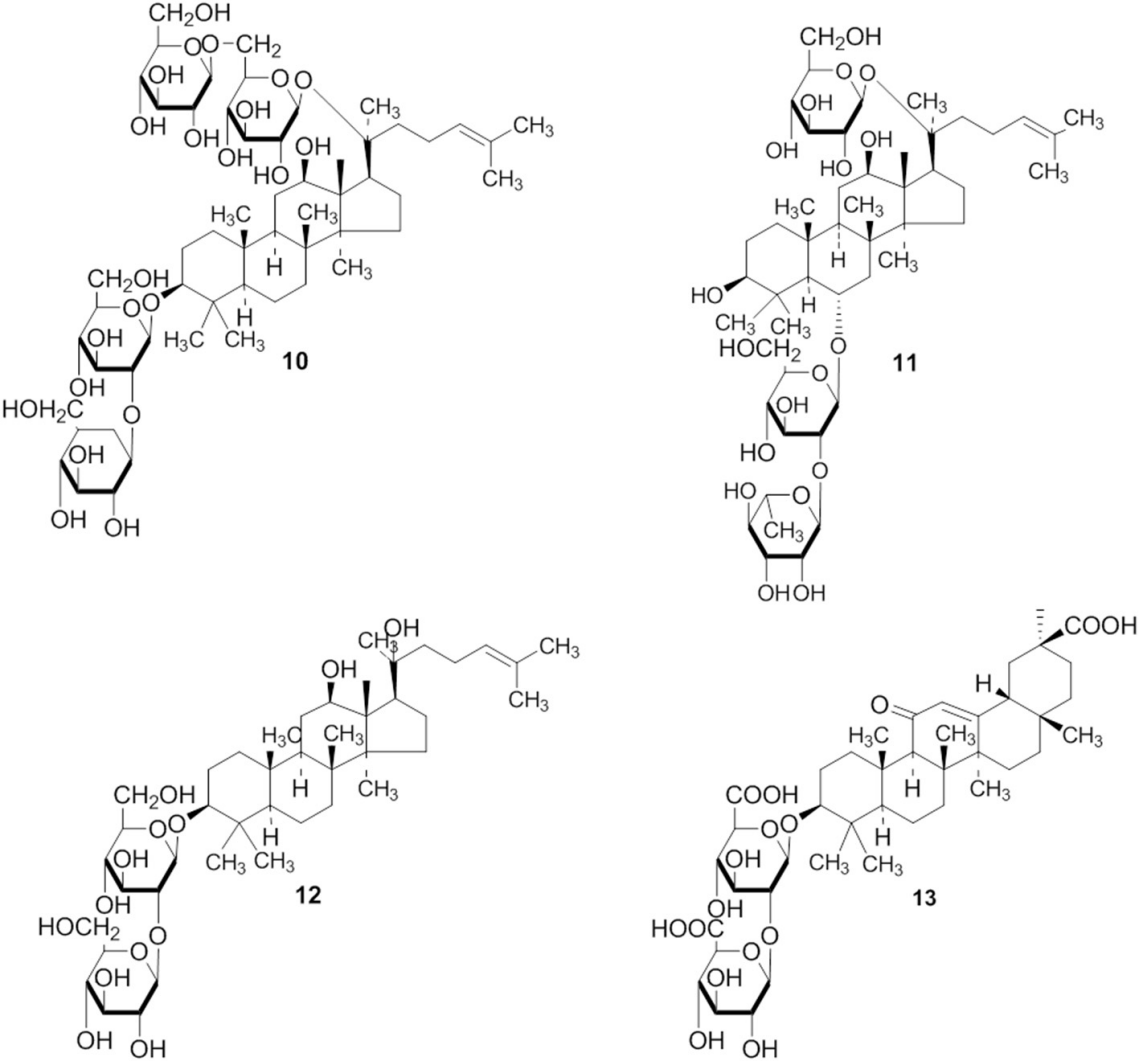
**The structure of 10 is corrected**.

**Figure 6:**
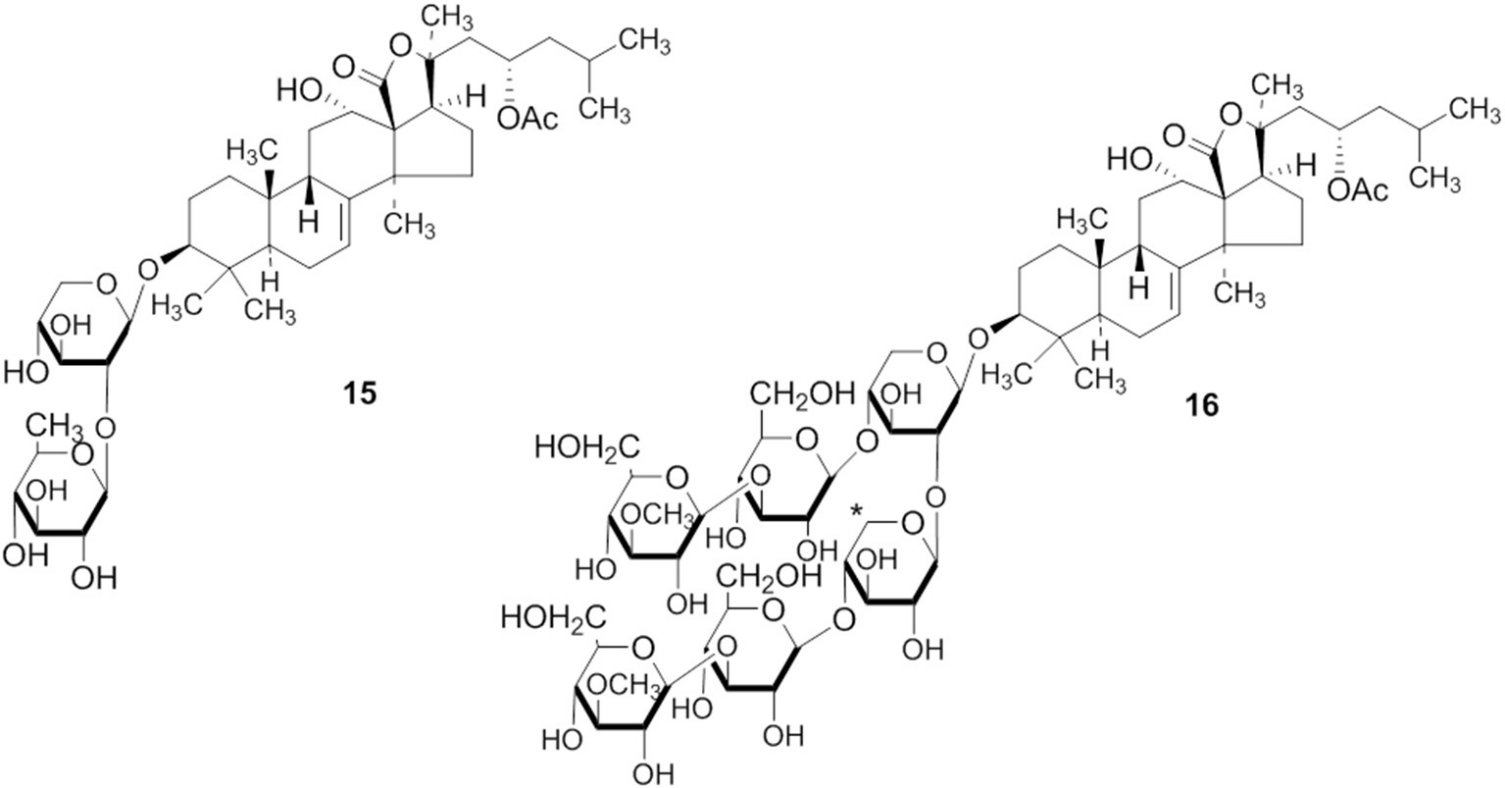
**The structure of 15 is corrected**.

**Figure 9:**
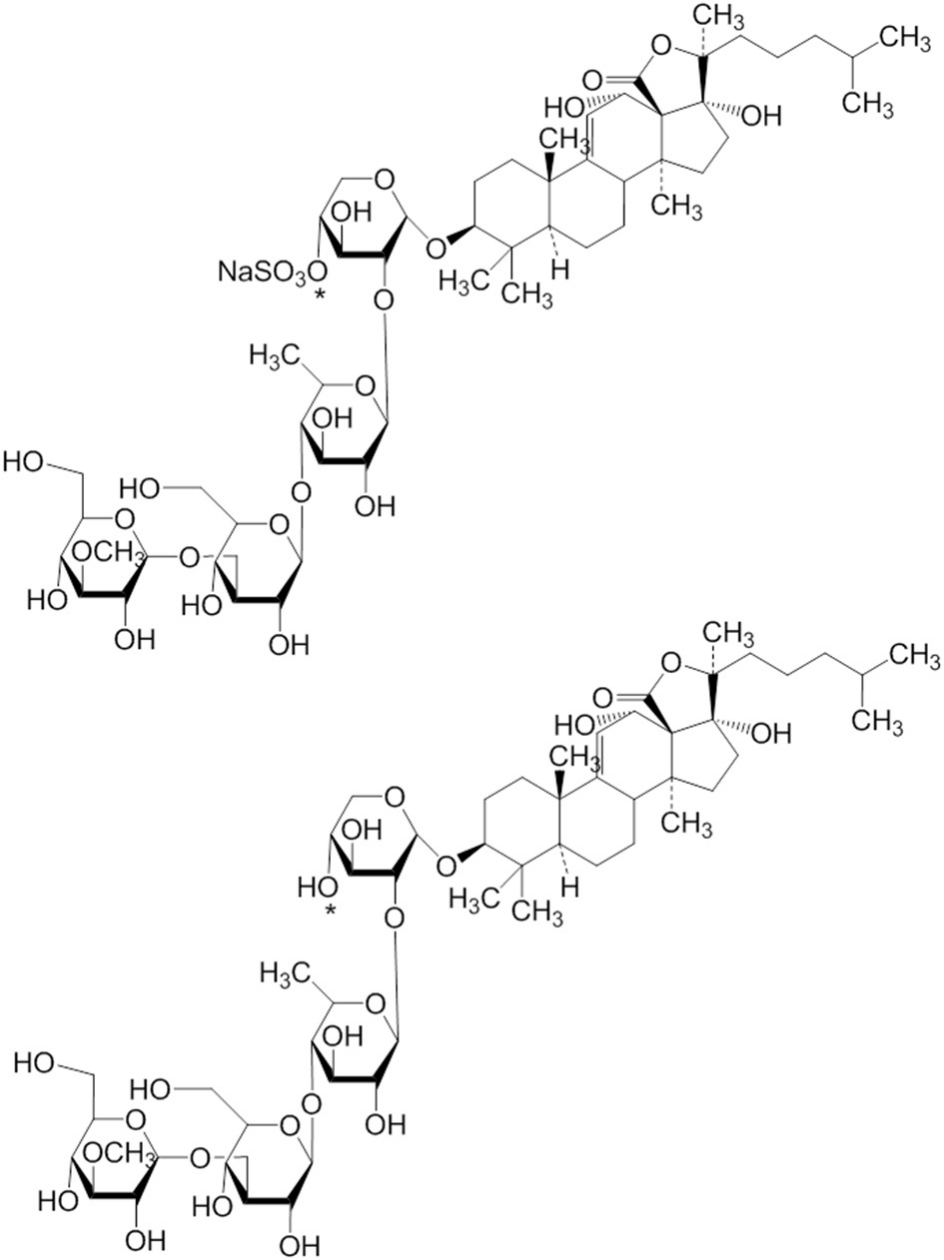
**The structures of 20 and 21 are corrected**.

1. The structures of compounds (3, 4, 5, 6, 10, 15, 20, and 21) are corrected.

2. Compounds 17 and 18 (in Figure 7) are same as compounds 20 and 21 (in Figure 9).

## Conflict of interest statement

The author declares that the research was conducted in the absence of any commercial or financial relationships that could be construed as a potential conflict of interest.

